# Brain Abscess

**Published:** 1907-05-25

**Authors:** Charles A. Ballance

**Affiliations:** M.V.O.; Surgeon to, and Lecturer on Surgery at, St. Thomas's Hospital; Surgeon to the National Hospital for the Paralysed, Queen Square


### HOSPITAL CLINICS.

### BRAIN ABSCESS.

By CHARLES A. BALLANCE, M.V.O., M.S., F.R.C.S., Surgeon to, and Lecturer on Surgery at, St. Thomas's Hospital; Surgeon to the National Hospital for the Paralysed, Queen Square.

(A Lecture delivered at the National Hospital for the Paralysed and Epileptic, Queen Square.)

### A SECONDARY NOT A PRIMARY DISEASE.

THIS afternoon we will discuss the subject of abscess of the brain. Abscess of the brain is a secondary and not a primary disease. In almost all cases it occurs as a complication of one or other of four conditions; these are (1) injury of the head; (2) local cranial suppuration; (3) certain general infections; (4) certain local suppurations other than those of the head. When secondary to head injury a brain abscess is not, as a rule, deeply-seated, but is a meningo-cortical abscess. The abscess wall is formed by the meninges and the superficial layers of the brain substance which have participated in the suppurative inflammation. The specimens on the table illustrate this condition—superficially placed brain abscess secondary to injury of skull. The instrument causing the injury might have penetrated deeply into the brain substance carrying infection with it like a stab-culture, then a deep-seated abscess would have resulted. More than half of all brain abscesses are due to the second of the causes I have named—local suppuration in the skull. The local cranial suppurations which determine brain abscess are either in the temporal bone or in the cavities connected with the nose. The site of infection of the brain substance from local cranial suppuration is, commonly, at the point where the brain tissue is most closely approximated to, or most intimately connected with, the suppurative focus in the bone. In temporal bone (ear) suppuration the parts of the brain nearest the temporal bone, the temporosphenoidal lobe and the cerebellum, are those most frequently affected.

### FREQUENCY OF SITUATION.

The cerebellar hemisphere lies behind the posterior surface of the petrous bone, and the temporosphenoidal lobe is close against the roof of the tympanum; thus it is easy to understand why these regions are so frequently infected from suppuration in the temporal bone. Brain abscess is much more likely to result from chronic than from acute local suppuration. Intra-cranial infection from an acute condition determines acute meningitis rather than brain abscess, because the sub-arachnoid space is then infected before limiting protective adhesions have had time to form. In chronic suppuration infection only slowly reaches the interior of the skull, protective adhesions form before the dura is traversed, and the infection, making its way through the fused meninges, reaches the brain substance without entering the general cavity of the arachnoid or the sub-arachnoid space. The highly vascular cortex, with abundant connective tissue corpuscles, offers considerable resistance to the spread of infection, and does not commonly undergo extensive destruction. Where it is traversed by the infective material a barrier of fibrous tissue is thrown out limiting the destructive process to the formation of a narrow track. The white matter is much less resistant and is, therefore, much more extensively destroyed. In this way the abscess assumes a pear-shaped form connected by a narrow portion or "stalk" of greater or less length with the diseased bone.

### CONDITIONS FAVOURABLE TO MANIFESTATION OF BRAIN ABSCESS.

The general infections most liable to be complicated with abscess of the brain are (*a*) pyæmia, (*b*) tubercle, and (*c*) certain specific fevers, such as influenza, enteric fever, and variola.

Brain abscess is a rare manifestation of general pyæmia, and when it does occur is usually on the left side of the brain. I shall refer later on to brain abscess complicating influenza.

The local suppurations elsewhere than in the skull which occasionally become complicated with brain abscess are caries of rib, empyæma, bronchiectasis, and gangrene of lung. That these suppurations in the chest do directly cause infection of the brain substance is proved by the fact that in some such cases lung pigment has been found in the pus of the brain abscess. The moment when infection reaches the brain is not commonly marked by any recognisable local symptom, and the course of brain abscess varies within wide limits.

### CLINICAL TYPES.

Five types of clinical evolution may be described.

1. Sub-acute evolution more or less distinctly divided into three stages: (*a*) The initial febrile stage; (*b*) the stage of remission; and (*c*) the paralytic stage. 2. Evolution with severe general infection. 3. Evolution with complete latency until the final attack of coma. 4. Evolution just like that of brain tumour. 5. The remittent type of evolution.

I. The sub-acute type is that we so often see in brain abscess secondary to ear disease or, less frequently, to disease in the accessory cavities of the nose. It generally lasts about three weeks, but sometimes longer. The initial symptoms are acute; the face is flushed, the temperature rises sharply, there is severe headache and vomiting; the patient is obviously ill. An acute specific fever seems a likely enough explanation of the symptoms, but attentive examination leads to the discovery of local cranial suppuration, and we should at once suspect brain abscess. This acute stage with severe headache is of variable, but usually of short, duration. At the end of three days, or thereabouts, the case passes into the second stage, that of remission, the temperature falls, and the headache greatly diminishes in intensity; the patient no longer calls out with severe pain in the head, but his mental state is dull. The disease has produced increase of the intra-cranial pressure; this has dulled sensation, and so the severity of the symptoms has passed away. On this condition the third or paralytic stage supervenes, usually suddenly. There is then manifest motor weakness, the muscles affected naturally varying with the site of the abscess; for instance, in abscess of the cerebellar hemisphere the muscular weakness will be most decided in the homo-lateral upper limb, and in those of the contra-lateral limbs in abscess of the temporo-sphenoidal lobe.

II. The second type of clinical evolution is exceedingly fatal. The onset is hyperacute, there is high fever, rapid pulse, often wild delirium. It is often quite impossible, with our present knowledge, to make a diagnosis of brain abscess from the symptoms present, and some cases of brain abscess with severe general symptoms have been taken for a malignant form of some acute specific fever, or the disease known as acute delirious mania.

III. In the third type of evolution there are practically no symptoms until the final stage; in this the patient suddenly becomes comatose and dies instantly or in a few hours. Many such cases are on record. Perhaps, in some, diagnosis is impossible, but it should always be remembered that symptoms not present and symptoms not observed are not synonymous terms, and in many of the recorded cases there is no evidence that the patient had been under skilled observation before the onset of coma. Some of the manifestations of gross disease of the brain cause the patient but little inconvenience, and are only to be elicited by an attentive clinical examination, and in some, at least, of the cases of "complete latency," it seems probable that such an examination would have revealed some symptom or symptoms from which a diagnosis might have been made.

IV. In the fourth type the clinical evolution is like that of brain tumour. The abscess is chronic and the onset of symptoms is gradual; just like those caused by any other slowly increasing growth in the brain. Headache, at first slight and occasional, later more severe and more constant, vomiting, and optic neuritis, are the general symptoms of such an abscess, to which after a few weeks the "focal" symptoms indicating the site of the abscess are gradually added. The abscess, in fact, causes just the same symptoms as a tumour of the same size, in the same situation, and growing at the same rate would cause.

V. The fifth, the remittent type, is very important. The patient has an acute illness of which all the symptoms subside, but at the end of two or three months or so they recur suddenly, and in a few days the patient may be dead. All cases of this type that I have seen have been influenzal, and I cannot but think that many cases of influenzal brain abscess have been unrecognised. The late Dr. Bristowe first described these cases. Each of his two patients had had influenza, during an epidemic, with high fever, very severe headache and great subsequent prostration; both, except that the headache in some degree persisted, got quite well, but after two or three months each of them became acutely and suddenly ill with severe headache and vomiting, and died within a few days. On post-mortem examination an encapsuled abscess about the size of an egg and filled with green pus was found in the brain in each case. In neither case was there aural or nasal or other local cranial suppuration, so that the abscesses must have been due to direct infection of the brain substance carried by the blood-stream. In one case the abscess was in the fronto-parietal, and in the other in the occipito-sphenoidal, region. Many years ago a somewhat similar case following influenza was in this hospital under the care of Dr. Taylor. Three or four months before admission the patient had a very severe attack of influenza during which some weakness of the left side of the body was observed. This paresis was entirely recovered from and was not present on admission. He had never been really well since the influenzal attack and complained much of occipital headache. Optic neuritis was well marked on admission, his temperature was 103, and there was urgent vomiting. The same evening he became comatose, and I was sent for between 9 and 10 p.m. to operate. Respiration had ceased before I arrived and artificial respiration was maintained. I consulted with Dr. Taylor as to the plan of operation. In the afternoon it had been thought that the symptoms pointed to an encapsuled cerebellar abscess. The left-sided weakness might have been due to a lesion in the homo-lateral cerebellar hemisphere or to one in the contra-lateral cerebral hemisphere, and no indications were at the time available for determining this point. As an operation in the cerebellar region would, during the performance of artificial respiration, be of great difficulty, I determined to expose the right motor region. I removed a piece of bone about four inches square, the brain bulged through the opening for about an inch, showing that it was under great pressure. Natural respiration did not, as we had hoped, return. A trocar and cannula was plunged up to the hilt into the brain and impinged upon a hard mass into which it would not penetrate. I felt sure that this was a tumour in an inaccessible part of the brain, and, therefore, abandoned the operation. Death occurred directly the artificial respiration was discontinued. The post-mortem examination revealed an encapsuled abscess containing an ounce of thick pus in the situation of the optic thalamus, the capsule was very dense and about a quarter of an inch thick. At the present day an incision would be made in the cortex, the finger passed in, and such an abscess, or tumour, enucleated.

### General Symptoms.

Of the symptoms to which brain abscess gives rise some indicate the presence of an intra-cranial lesion, but give no information as to its site, while others indicate a lesion of a particular part of the brain; we therefore speak of general cerebral symptoms and localising or focal cerebral symptoms. The first group, the general symptom-complex, or syndrome, is due in the main to increased intracranial pressure. The symptoms in this group are headache, vomiting, optic neuritis, slow pulse, slow respiration. From these symptoms alone, no localisation diagnosis can be made. Pain, even when referred constantly to a particular spot, does not indicate the site of the lesion. Persistent localised pain, however, when associated with persistent localised tenderness is of some localising value. Optic neuritis is often more intense or is observed earlier on the side of the lesion.

The localising or focal symptoms are due to irritation of, or suppression of function of, a particular part of the brain. For their correct interpretation a considerable knowledge of cerebral physiology is required, and several lectures would be needed for their complete discussion—a few only can now be considered.

### Localising Symptoms.

The localising symptoms are of two kinds: (*a*) Abnormal motor phenomena, and (*b*) abnormal sensory phenomena. I will give you a few illustrations of the association of such symptoms as observed in abscess of particular parts of the brain.

*Abscess in Cerebellar Hemisphere.*—In some, though not all, cases of abscess in the cerebellar hemisphere, the patient assumes a "forced position" in bed. He lies curled up on one side with the elbows and knees flexed and the side of the face corresponding to that of the lesion uppermost. I have seen this attitude adopted in two or three cases of cerebellar tumour; the same position is assumed in experimental ablation of the cerebellar hemisphere, and has been observed in some cases of tumour. Deviation of eyes: Usually conjugate deviation of both eyes away from the side of the lesion, but sometimes skew deviation with divergent optic axes. Lateral nystagmus, most marked when the patient looks towards the side of the lesion. A very characteristic symptom of lesion of the cerebellar hemisphere, abscess or tumour, is weakness and loss of tone of the muscles of the homolateral limbs, the upper limb especially.

I once operated upon a case diagnosed as left cerebellar tumour; no tumour was found, but the cerebellum bulged through the opening in the dura under very great pressure. So great was the intracranial pressure that most of the cerebellar hemisphere continued to protrude and ultimately sloughed off. For many months the upper limb on the same side was almost paralysed. The patient lived some eighteen months, and then died after a short illness; at the post-mortem a tumour was found in the right frontal lobe. It is sometimes difficult to distinguish a frontal lobe tumour from a cerebellar tumour.

When we have reason to suspect an abscess in the cerebellar hemisphere and we find decided weakness in the arm on the same side, this symptom is pathognomonic, and operation should be done at once. In some few cases rigidity and spasm have also been observed on the homo-lateral side. A case of cerebellar abscess was reported some years ago by Deansely (of Wolverhampton) in which there was paralysis of both arm and leg on the side of the lesion. The knee-jerks may be either diminished or exaggerated; it is not uncommon to find the knee-jerk exaggerated on the homo-lateral side. In some cases of cerebellar abscess the curious phenomenon known as forced rotation is present. In lesions of the cerebellar hemisphere the rotation is round a vertical axis away from the lesion. In lesions of the vermis rotation is round a horizontal axis, as shown in Pagano's experiments. In a case of right-sided cerebellar abscess that I saw some years ago the patient had a tendency to rotate away from the side of the lesion and to fall towards the side of the lesion. He came to the hospital in the fifth week of the disease and was still able to walk, though with considerable difficulty, always tending to fall towards the side of the lesion. Mills, of Philadelphia, an eminent neurologist, says that in these cases the tendency to fall is invariably towards the side of the lesion. I do not know that this rule is absolute, but Dr. Mills is so great an authority that one does not care to contradict him. Lesions of the cerebellum cause no sensory phenomena, all forms of sensation remain intact. But abscess of the cerebellum is often associated with deafness; the deafness is due, not to the cerebellar abscess, but to the disease in the temporal bone; it is deafness from destruction of the peripheral sense organ, and is, therefore, on the side of the lesion. The syndrome symptoms in cerebellar abscess present peculiarities which are worthy of mention, as they are often of diagnostic value. Optic neuritis occurs early, and is manifest in the eye on the same side as the abscess, earlier than it is in the eye on the opposite side, so that optic neuritis limited to, or more advanced in, one eye is probably diagnostic of the side of the lesion. Vomiting is urgent and vertigo intense, much more so than in similar lesions of the cerebrum.

*Temporo-sphenoidal Abscess.*—Let us now consider, in the same way, the symptoms of abscess of the temporo-sphenoidal lobe. The abnormal motor phenomena are (1) paralysis of the third nerve, on the side of the lesion, from direct pressure; it is first manifested by immobility of the pupil, though it may go on to paralysis of the oculo-motor muscles supplied by the third nerve. With ear disease and the general symptom of cerebral abscess a stabile pupil on the side of the lesion is diagnostic of abscess in the temporo-sphenoidal lobe. (2) More or less complete hemiplegia. This is on the side opposite to the lesion and is due to the abscess extending to, and destroying the motor cortex, or pressing on the internal capsule. The march of the paralysis differs in the two cases: in the internal capsule type of hemiplegia the leg is first affected, then the arm, and lastly the face; when the hemiplegia is due to gradual extension of the abscess up the cortex, the face is first affected, then the arm, and lastly the leg. Thus the patient, when first seen, may have slight weakness of the opposite side of the face, next day the arm may be weak, and a little later the leg, or the onset may be in the reverse order. (3) Exaltation of the deep reflexes on the contra-lateral side.

The abnormal sensory phenomena are: (1) Deafness of the opposite side from extension of the abscess to the cortical centre for hearing which, as Ferrier long ago demonstrated, is located in the superior temporo-sphenoidal convolution. It should be remembered that the disease of the temporal bone may destroy the organ of hearing and so cause deafness on the side of the lesion, and that the cortical centre for hearing, though frequently, is not necessarily involved in a temporo-sphenoidal abscess, so that with a temporo-sphenoidal abscess there may be deafness on the homo-lateral, on the contra-lateral, or on both sides, but the deafness caused directly by a temporo-sphenoidal abscess is always contra-lateral.

2. The cortical centres for taste and smell are sometimes affected in temporo-sphenoidal abscess.

3. More or less complete anæsthesia on the contra-lateral side when the abscess has extended towards the internal capsule, or only some loss of the power of localising light touches and loss of muscular sense when the cortex only is affected.

4. In abscess of the left temporo-sphenoidal lobe in right-handed individuals, or of the right in left-handed individuals, speech defects are due to disturbance of the cortical centres concerned in articulate speech. These important symptoms make the diagnosis of left-sided temporo-sphenoidal abscess much easier than that of a similar abscess on the right side. I have met with many cases of temporo-sphenoidal abscess in which aphasia was present. One such case was in St. Thomas's Hospital fifteen years ago. He had left ear disease, but did not seem gravely ill, though he complained of persistent headache. Two or three days after his admission the sister told me that he had been asking her during the morning to boil a sixpence; from that I knew that he had aphasia, and that consequently the temporo-sphenoidal lobe was involved. It was ascertained upon inquiry that the patient had been in the habit of giving his wife sixpence with which to buy eggs. A large temporo-sphenoidal abscess was evacuated the same day.

5. Another important sensory phenomenon of temporo-sphenoidal abscess, whether left or right, is the peculiar mental condition now described by Dr. Hughlings Jackson as the "dream state," and by Sir Wm. Macewen as the "somnambulistic state." In it the patient is drowsy and apparently unconscious of his surroundings, and the eyes seem staring into vacancy. The phenomenon is especially characteristic of abscess of the anterior part of the temporo-sphenoidal lobe.

*Frontal lobe Abscess.*—I will now say a few words to you about abscess of the frontal lobe. The danger attending chronic aural suppuration is now fairly generally appreciated by the profession, but the equally great danger of chronic nasal suppuration seems less clearly understood. Many preventable deaths from intra-cranial infection still result from chronic nasal suppuration. Obviously chronic nasal suppuration ought to be as thoroughly dealt with as aural suppuration. When this disease gives rise to intra-cranial infection it is usually by way of the frontal sinus. Meningitis, or brain abscess, may result. Frontal lobe abscess has usually an insidious course. It is not uncommon to find a thick-walled encapsuled abscess in the frontal region.

The symptoms met with in frontal lobe abscess are alteration, especially deterioration, of mental character, incontinence of urine, and deviation of head and eyes from irritation or destruction of the centre for movement of head and eyes in the second and third frontal convolutions. On the left side these centres are situated between, and in advance of, Broca's speech-centre in the third, and Charcot's motor graphic centres in the second, frontal convolution.

In an abscess extending backwards there would be symptoms due to interference with the motor centres in the ascending frontal convolution, and on the left side, also, with the speech centres.

### The Treatment.

The treatment of cerebral abscess is complete evacuation and drainage. Whenever possible, the abscess should be opened and drained through its "stalk," that is through that part which is connected directly with the focus of bone suppuration to which the abscess owes its origin. The local bone disease should be first removed and the operation continued by following its track right into the abscess. This method is far preferable to that which was formerly practised. In certain urgent circumstances, such as arrest of respiration, the old plan in which the abscess is opened through healthy cortex and meninges is still necessary.

